# A Compact Inductive Position Sensor Made by Inkjet Printing Technology on a Flexible Substrate

**DOI:** 10.3390/s120201288

**Published:** 2012-01-31

**Authors:** Nikola Jeranče, Dragana Vasiljević, Nataša Samardžić, Goran Stojanović

**Affiliations:** Faculty of Technical Sciences, University of Novi Sad, Trg Dositeja Obradovića 6, Novi Sad 21000, Serbia; E-Mails: njerance@uns.ac.rs (N.J.); vdragana@uns.ac.rs (D.V.); nsamardzic@uns.ac.rs (N.S.)

**Keywords:** angular position sensor, flexible meander, Kapton film

## Abstract

This paper describes the design, simulation and fabrication of an inductive angular position sensor on a flexible substrate. The sensor is composed of meandering silver coils printed on a flexible substrate (Kapton film) using inkjet technology. The flexibility enables that after printing in the plane, the coils could be rolled and put inside each other. By changing the angular position of the internal coil (rotor) related to the external one (stator), the mutual inductance is changed and consequently the impedance. It is possible to determine the angular position from the measured real and imaginary part of the impedance, in our case in the frequency range from 1 MHz to 10 MHz. Experimental results were compared with simulation results obtained by in-house developed software tool, and very good agreement has been achieved. Thanks to the simple design and fabrication, smaller package space requirements and weight, the presented sensor represents a cost-effective alternative to the other sensors currently used in series production applications.

## 1. Introduction

Rotation position sensors are widely used in modern automotive and industrial applications [1]. Most common requirements are robustness, low cost, insensitivity to dirt, high temperature range, variable stroke, no wear-out. Inductive sensors are known to be a sensible choice for such applications [2] particularly from the point of view of temperature independence and insensitivity to mechanical tolerances (effective in the engine compartment). An inductive position sensor that consists of a thin coil wound around the actuator for microrobotic applications has been already presented [3], but it was manufactured combining three costly technologies (Micro Electro Discharge Machining, Deep Reactive Ion Etching and micromilling) and lacked the feature of mechanical flexibility. A linear displacement sensor based on the inductive concept was described in [4], using a copper meander coil and pattern guide made from a soft iron. A hydrogel-based passive wireless sensor with a micromachined inductive transducer was investigated in [5], but it was not a position sensor and the sensor was designed for biomedical/chemical sensing applications. The transducer utilized the dependence of mutual inductance on the gap separation between two planar spiral coils, microfabricated using copper-clad polyimide film (commonly used for flex-circuit manufacturing). The noncontacting type, angular position inductive sensors were already analyzed, but for very specific applications such as underground boring tools [6,7].

In recent years, there has been tremendous interest in the development of printed electronics components as a means of achieving ultra-low-cost and ubiquitous electronic circuits. While there have been several demonstrations of printed organic transistors or OLED or RFID up to now, there has been a few published papers about passive components fabricated using inkjet technology on flexible substrates [8–10] and their applications in different sensors.

In this paper, an inductive angular position sensor made on a flexible substrate is proposed. The sensor is composed of one internal coil (acting like a rotor) and one external coil (acting like a stator). The silver coils (in meander geometry) are firstly printed using ink-jet technology on Kapton film, in the plane, and after that rolled in the air (without ferromagnetic cores). The outside diameter of the sensor was 11.4 mm and the length was 12 mm (aspect ratio = 1.05). In this way, we have avoided the necessity to wind wire (usually copper) for coils, to include any magnetic materials and to make costly metallic parts in circular shape to be a platform for stator and rotor part of the sensor. Other important advantages of the proposed printed sensor are: mechanical flexibility, light weight, short design and fabrication time, low fabrication cost, absolute reproducibility, compact design and wide range of applicability. Apart from this, printed sensors have a number of ecological benefits such as the efficient use of materials, environmental friendly manufacturing and power efficiency. Furthermore, it is important to note that proposed sensor can be integrated into the shape of any object or curved surface (the property of formability).

## 2. Sensor Structure and Sensing Principle

**Figure 1.**
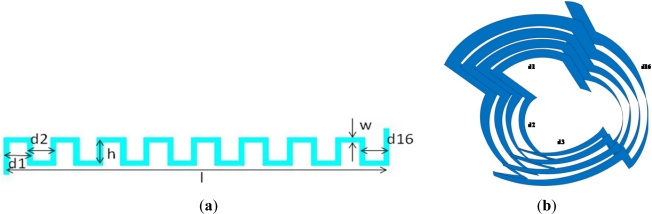
Designed meander inductor for internal sensor’s coils—(**a**) unrolled, in the plane and (**b**) rolled, 3D.

The sensor consists of two spirally rolled meander inductors printed on flexible substrate. An unrolled meander inductor is drawn in Figure 1(a); by rolling it, the structure in Figure 1(b) is obtained.

The presented sensor is composed of two such meanders, spirally rolled around the same axis. One of them (rotor) is able to rotate around this axis, while the other one (stator) is fixed. The rotor meander has 4 turns with the following dimensions: width w = 2 mm, distances ranging from d1 = 7.95 mm to d16 = 8.31 mm, the height h = 10 mm and the length l = 129.44 mm.

The overall occupied area of the rotor coil, in the plane (unrolled), was (l + w) × (h + w) = 1,577.12 mm^2^. The stator meander has also four turns with the following dimensions: width w = 2 mm, distances ranging from d1 = 8.58 mm to d16 = 8.94 mm, the height h = 10 mm and the length l = 139.49 mm. The overall occupied area of the stator coil, in the plane (unrolled), was (l + w) × (h + w) = 1,697.88 mm^2^. When the stator and rotor meanders are rolled, for each turn the radius is increased by substrate’s thickness, therefore we can write the radius depending on the angle of spiral rolling *θ*:

(1) $$r=r_{0}+a\;\theta$$

where *r*—radius, *r_0_*—radius at the beginning, *a—*constant determined by *a* = *t*/2π, where *t* is a substrate thickness. Parameters d1–d16 are 90 degree arc lengths corresponding to this radius increase, and they have been calculated according to Equation (1), using the formula:

(2) dl=rd θ

where *dl* is the differential of the arc length. The integration of Equation (2) is performed numerically by a computer program.

**Figure 2.**
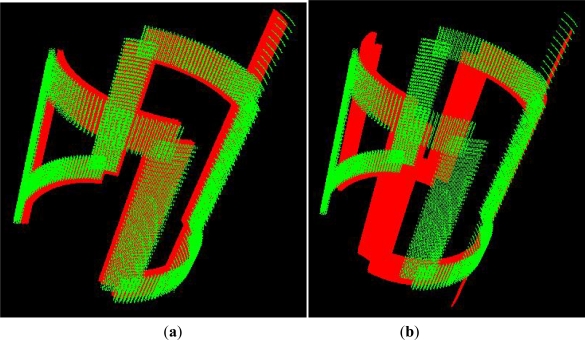
Two different angular positions of the rotor, (**a**) when the vertical conductive segments of the rotor coils are parallel and just above the same segments of the stator coil; (**b**) another angular position of the rotor coil—it corresponds to 30 degrees.

The mutual inductance of the two inductors varies with their relative angular position, for example in Figure 2 two different angular positions are illustrated. Alternating current flows through the excitation inductor. An electromagnetic field is generated which surrounds the rotor’s conductive segments. This causes alternating current to flow through the rotor as well, which in turn generates an electromagnetic field with a reaction on the receiving coils. Voltage is induced in the stator coil which depends on the position of the rotor and can be evaluated by further electronics (usually based on the microcontrollers). Thanks to its straightforward design, the sensor can be integrated easily and thus implemented economically in the various applications.

Both meanders (for stator and rotor coils) have been rolled in such a way that conductors with the same direction of the current are next to each other. Following this principle the maximum of the mutual positive inductance would be obtained. The number of periods per turn determines the stroke of the sensor. In this paper, a sensor with 90 degree stroke is described.

This particular stroke is chosen because it is frequently used in automotive and industrial applications (in valves, for example). When the rotor is displaced with respect to the stator, the displacement angle can be measured by periods of 90 degrees, reproducible for every turn. In order to prove the sensing principle, the measurements for several positions in one 90 degree stroke have been done, with the rotor manually displaced with respect to the stator.

## 3. Design and Fabrication of Inductive Components

The width w of the conductors (see Figure 1) is adopted to be 2 mm, in order to decrease the resistance as much as possible for the chosen sensor dimensions. For rotor meander, the spiral (rolled coil) starts at radius 5 mm and 0.075 mm is added at each full turn. The stator meander is rolled starting at 5.4 mm. All the other parameters are the same as for the rotor.

### 3.1. Simulation Results

Before printing, a designed sensor structure was verified by self- and mutual inductance calculation, using an in-house developed computer program [10], which calculates inductances by integrating through the currents. Values of the stator and the rotor self-inductance, obtained using our software tool, were 546 nH and 514 nH, respectively. The calculated mutual inductance values are given in Table 1.

**Figure 3.**
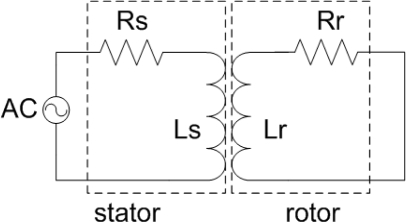
Schematic drawing of the sensor with short circuit at the rotor.

If the short circuit is made at the rotor, such as illustrated in Figure 3, the induced voltage on the rotor will generate current in the rotor circuit, which will induce voltage in the stator circuit. The measurements for several positions in one 90 degree stroke have been done, with rotor manually displaced to match the previously marked positions on the stator.

The voltage equations for the stator and rotor circuit can be written as follows:

(3) $$U_{S}=R_{S}\;I_{S}+j\omega L_{S}\;I_{S}+j\omega {\mathrm{MI}}_{R}$$

(4) $$0=R_{R}\;I_{R}+j\omega L_{R}\;I_{R}+j\omega {\mathrm{MI}}_{S}$$

where *U_S_*—stator coil voltage, *I_S_*—stator coil current, *I_R_*—rotor coil current, *R_S_*—stator coil resistance, *L_S_*—stator coil self inductance, *L_R_*—rotor coil self inductance, *M*—mutual inductance between stator and rotor coils. The complex impedance *Z* (which can be written in the form *U_S_*/*I_S_*) is obtained by replacing *I_R_* from Equation (4) to Equation (3) and dividing Equation (3) by *I_S_*:

(5) $$Z=R_{S}+j\omega L_{S}+\frac{\omega^{2}\;M^{2}}{R_{R}+j\omega L_{R}}$$

Separating the real (*Z_real_*) and the imaginary part (*Z_imag_*) of the impedance, the following expressions can be obtained:

(6) $$Z_{\mathrm{real}}=R_{S}+\frac{\omega^{2}M^{2}R_{R}}{R_{R}^{2}+\omega^{2}L_{R}^{2}}\;\;\;\;\;Z_{\mathrm{imag}}=\omega L_{s}-\frac{\omega^{3}M^{2}L_{R}}{R_{R}^{2}+\omega^{2}L_{R}^{2}}$$

**Figure 4.**
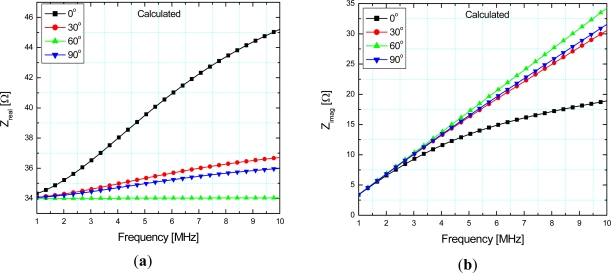
Calculated (**a**) real part–*Z_real_*; (**b**) imaginary part–*Z_imag_* of the stator impedance with the short circuit at the rotor depending on frequency and angular position.

The electric circuit equations have been solved for the impedance measured at the ends of the sensor and its real and imaginary part are given in Figure 4(a,b), respectively, using the measured values of the stator and the rotor electrical resistance equal to 34 Ω and 23.5 Ω, respectively. The impedance is given as a function of frequency while angular position of the sensor (0°, 30°, 60° and 90°) is used as a parameter.

As can be seen from Figure 4(a) a declining order of curves for 0, 30, 90 and 60 was obtained, whereas in Figure 4(b) a decreasing order was obtained for 60, 90, 30, 0 degrees. These curves are obtained from the expressions for *Z_real_* and *Z_imag_* at different frequencies (Equation (6)), with *M* as a parameter changing with different angular positions. The irregularity in these curves is a consequence of different values of mutual inductance (*M*) between the coils. The position of the rotor can be determined through the values of either *Z_real_* or *Z_imag_* at one frequency.

An important advantage of our sensor is that the proposed construction does not use any magnetic material. The magnetic core, even if made from the best ferromagnetic material, introduces to the transfer function of the sensor some nonlinear factors which depend on temperature, frequency, flux density, *etc*. Additional magnetic noise also decreases the resolution of the sensor [11].

### 3.2. Fabrication of the Sensor

**Figure 5.**
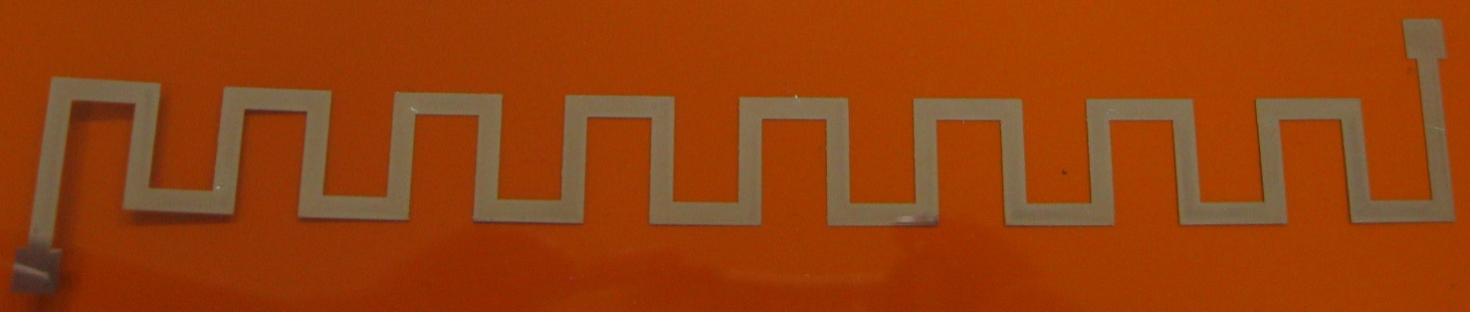
Printed unrolled rotor inductor.

The designed inductive sensor components were fabricated using a Dimatix DMP-3000 materials deposition printer [12]. The resolution of the inkjet process using this machine is mainly governed by the nozzle diameter (approximately the droplet diameter) and the statistical variation of the droplet flight and spreading on the substrate. In case of printing with silver nanoparticle ink (Suntronic Jet Silver U6503) [13], the minimum droplet diameter was around 50 μm, and drop spacing was 25 μm (from center to center) obtained by changing the printhead angle. Inductors were printed using silver ink on Kapton film [14] substrate, 75 μm thick and with dielectric constant of 3.2. The printed inductor for the rotor part is shown in Figure 5. The meander inductors were chosen because of the simple, one metal layer structure (fast fabrication), and rolled them (thanks to the flexibility of the substrate) we can obtain multilayered structures. The weight of one rolled inductive sensor part was approximately 1.5 g (this is also one of the advantages of our sensor).

**Figure 6.**
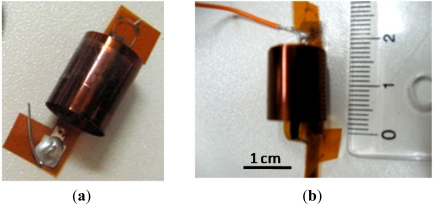
(**a**) the rolled rotor inductor; (**b**) the final structure of the sensor with dimensions.

After printing and drying (in the oven, for 45 minutes at the temperature of 200 °C), the substrates with meander conductors have been carefully rolled and the rotor was put inside the stator. Rolled rotor meander and final sensor structure are given in Figure 6(a,b), respectively.

## 4. Measurement Results and Discussion

**Figure 7.**
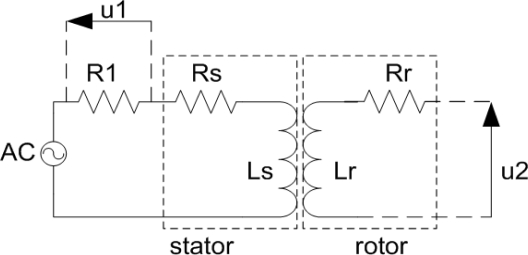
Illustration of the equivalent circuit for the mutual inductance measurement.

The measurement of mutual inductance for different angular positions have been performed in two steps: first, an AC voltage was applied to the stator and the input current of stator was measured, using equivalent circuit given in Figure 7, and then the induced voltage u2 was measured on the open rotor circuit, for the same input AC voltage.

**Figure 8.**
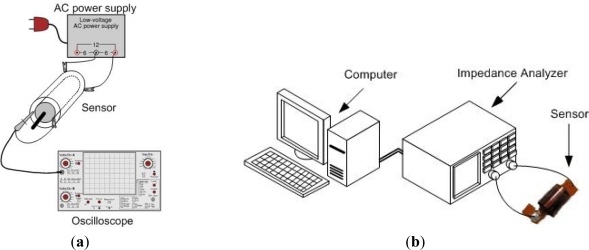
Illustration of the experimental setup for measuring (**a**) mutual inductance and (**b**) impedance.

A resistance R1 (47.8 Ω) is connected in series with stator, and the voltage drop u1 on R1 is measured, in order to obtain the input current. The measurements have been done at 7.5 MHz frequency of AC voltage. The experimental setup for the performed measurements is presented in Figure 8. Figure 8(a) illustrates measuring process of mutual inductance–*M*, whereas Figure 8(b) depicts experimental setup for impedance measurement.

In this measurement, we cannot see the phase of the induced voltage relative to the stator current, therefore we can only obtain the absolute value of the mutual inductance by the following expression:

(7) $$M=\frac{R_{1}u2}{2\pi fu1}$$

The Equation (7) has been used as a verification step to obtain the mutual inductance from measured induced voltage with open rotor circuit for different angular positions at one frequency of stator input voltage. It provides an efficient way to calculate the angular position directly, if the sensor can be made in such a way that the induced rotor voltage is accessible. In other version of the sensor, the rotor circuit is closed (short-circuit) and the variation of mutual inductance *M* can be followed as changes of *Z_real_* and *Z_imag_*. The measurement results for the mutual inductance are given in Table 2 and they are in very good agreement with the calculated inductance values given in Table 1.

**Figure 9.**
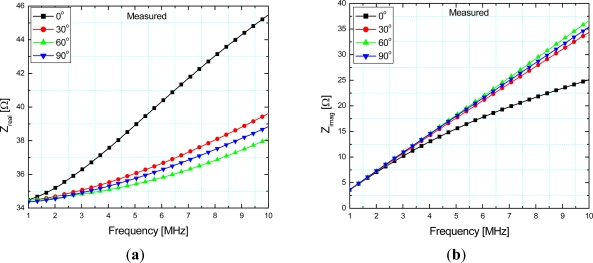
Measured (**a**) real part–*Z_real_*; (**b**) imaginary part–*Z_imag_* of the stator impedance with the short circuit at the rotor depending on frequency and angular position.

**Figure 10.**
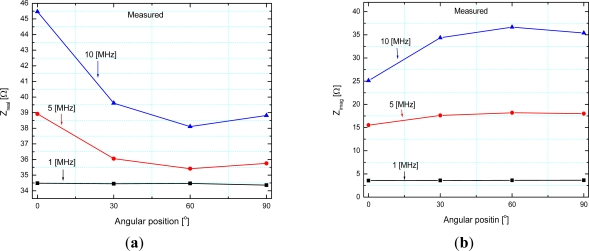
Measured (**a**) real part–*Z_real_*, (**b**) imaginary part–*Z_imag_* of the stator impedance with the short circuit at the rotor as a function of the angular position.

The rotor was positioned according to previously made marks on the stator, corresponding to certain angles, and after that the measurements were done. The measured stator and rotor self-inductance were 573 nH and 490 nH, respectively. Measured stator and rotor resistance were 34 Ω and 23.5 Ω, respectively. This difference is a consequence of slightly longer conductive segments of the stator coil. The resistance values are not so high bearing in mind that silver is used as a functional material for meander conductive segments. The electric circuit shown in Figure 3 represents the sensor with the short circuit on the rotor. In this configuration, the impedance at the ends of the stator has been measured. The measured real and imaginary part of the impedance as a function of frequency are presented in Figure 9, whereas Figure 10 shows the measured real and imaginary part of the impedance as a function of the angular position of the rotor coil related to the stator coil.

As can be seen from the Figure 10, the real part of impedance (∼resistance) increases with increasing frequency due to the skin effect, which is more pronounced at higher frequencies. We can notice that both real and imaginary parts of the impedance are monotonous from 0 to 60 degrees. As a consequence of this, for a 90 degree stroke the rotor position is obtained from the value of mutual inductance (by measuring the induced rotor voltage–the sensor electric circuit is made according to Figure 7). If the stroke is limited to 60 degrees, it is possible to make a sensor with short circuit on the rotor (according to Figure 3), in which case only one of these values (*Z_real_* and *Z_imag_*) is sufficient to accurately determine the rotor position. The measured values of mutual inductance are in very good agreement with the calculated ones. The behavior of stator impedance with the short circuit at the rotor obtained by measurement at different frequencies can be predicted very well. Discrepancies in some values for the real part of the impedance can be explained by eddy currents (more pronounced for higher frequencies), which have not been taken into account in our software tool.

The use of the proposed sensor is limited by the following aspects: high temperatures (>200 °C) can deform or melt down the plastic substrate, the sensor needs processing of a non-linear input which can limit its dynamic response or cause an additional cost to the sensor, encapsulation and packaging is needed in order to protect sensor from harsh environments or the presence of ferromagnetic materials in the proximity of the sensor.

## 5. Conclusions

In this paper, a new inductive rotary position sensor structure has been presented. The sensor components were fabricated by printing sliver ink on a flexible substrate (Kapton film) using a Dimatix DMP-3000 materials deposition printer. This printer is a cartridge based piezo-ink jet printing system which enables direct deposition of fluids (functional materials) and offers easier, faster and less expensive product development for sensing applications. The foil-based sensor for 90 degree stroke was proposed. Calculated values of mutual inductances have been confirmed by measurements at different angular positions. The stator impedance for the short circuit at the rotor has been measured at different angular positions and position measurement principle has been confirmed. This sensor allows a simple measurement of rotary position with a compact sensor structure. Possible applications of the described sensor are the automotive industry, robotic industry, biomedical industry, *etc*.

## Figures and Tables

**Table 1.** Calculated mutual inductance for different positions of the rotor coil related to the stator coil.

**Position (°)**	**Mutual inductance (nH)**

0	439
15	342
30	216
45	121
60	25
75	−101
90	−185

**Table 2.** Measurement results with the open rotor–induced voltage and absolute value of mutual inductance.

**Position (°)**	**Measured amplitude of induced voltage (mV)**	**Measured absolute value of mutual inductance (nH)**	**Calculated mutual inductance (nH)**	**Relative difference**

0	260	440	439	0.22%
30	120	203	216	−6%
60	15	25	25	0%
90	130	220	−185	18.91%
